# Comparing Current Therapeutic Modalities of Androgenic Alopecia: A Literature Review of Clinical Trials

**DOI:** 10.7759/cureus.42768

**Published:** 2023-07-31

**Authors:** Parth S Bajoria, Prathma Anandbhai Dave, Ralph Kingsford Rohit, Charu Tibrewal, Naisargi Shrikant Modi, Siddharth Kamal Gandhi, Priyansh Patel

**Affiliations:** 1 Department of Internal Medicine, Gujarat Medical Education and Research Society Gandhinagar, Gandhinagar, IND; 2 Department of Internal Medicine, Medical College Baroda, Vadodara, IND; 3 Department of Internal Medicine, Dayanand Medical College & Hospital, Ludhiana, IND; 4 Department of Internal Medicine, Civil Hospital Ahmedabad, Ahmedabad, IND; 5 Department of Internal Medicine, M.P. Shah Government Medical College, Jamnagar, IND

**Keywords:** androgenic alopecia, treatment choices, drug therapy, minoxidil, finasteride, dutasteride, hair transplant

## Abstract

Androgenic alopecia (AGA), commonly known as male pattern baldness (MPB), is a hereditary condition characterized by hair follicles that are sensitive to androgens. This article focuses on examining the recent advancements in the comprehension and management of AGA. The genetic factors and pathophysiology of AGA, including the role of dihydrotestosterone (DHT) and the androgen receptor gene, are discussed. The consequences of hair loss on self-esteem and identity, as well as on mental health, are examined. Diagnostic methods, such as the hair-pull test and trichoscopy, are discussed. The article also presents the Hamilton-Norwood classification, which is the most commonly employed system for classifying MPB. The article then delves into the various treatment options available, including topical minoxidil, oral finasteride, platelet-rich plasma therapy, low-level light therapy, hair transplant, and other alternative treatments. The efficacy and combination therapies for these treatments are examined. Additionally, emerging treatments such as caffeine-based solutions and prostaglandin inhibitors are discussed. By examining the recent advancements in AGA treatment, this article provides a comprehensive overview for healthcare professionals to make informed decisions when selecting the best treatment options for their patients.

## Introduction and background

Androgenetic alopecia (AGA), also known as male pattern baldness (MPB), although it is seen in females too, is a hereditary condition characterized by hair follicles that are prone to shrinking in response to androgens. This primarily occurs at the crown of the scalp [1]. Numerous studies have suggested that elevated levels of dihydrotestosterone (DHT) and excessive expression of the androgen receptor gene are linked to the pathophysiology of AGA. Moreover, an enzyme called 5α-reductase (5-AR), found in the dermal papilla, plays a critical role in converting testosterone, both from the bloodstream and within the hair follicle, into DHT. DHT then binds to the androgen receptor within the hair follicle, leading to the activation of signaling pathways associated with ongoing miniaturization and eventual hair loss [2]. The pattern of hair loss varies in both sexes. In males, it is called male pattern hair loss (MPHL), and it involves a gradual thinning of hair on the vertex and frontal regions of the scalp, accompanied by a receding hairline. In females, it is called female pattern hair loss (FPHL), which presents with gradual thinning and hair loss in the frontal and vertex of the scalp without the frontal hairline receding [3]. The hair on the scalp holds significant symbolic value in defining a person's appearance and plays a crucial sociocultural role. Hairstyles and hair lengths are distinctive elements of an individual's identity, and the experience of hair loss can result in dissatisfaction with one's appearance, particularly among women, and can significantly impact self-esteem. In men, hair loss is also associated with psychological and social challenges [4]. Topical minoxidil and oral finasteride are widely considered the primary treatments for MPHL. For FPHL, the initial recommended treatment is typically topical minoxidil. However, apart from these approved treatments, there are several other pharmacologic and nonpharmacologic options available, although they may be considered off-label. These alternative treatments are used in certain cases and can include various medications or therapies aimed at promoting hair growth or preventing further hair loss [5]. Multiple studies have been carried out to decipher the best treatment for AGA. However, it is a big challenge for doctors to select the best treatment for their patients due to the lack of knowledge of the adverse effects and the potency of different modalities. In this article, we aim to review the most promising treatments that have made a breakthrough in the treatment of AGA in the last 10 years.

Methodology

Working in collaboration with other authors of this paper, we searched articles on PubMed Central, MEDLINE, and Google Scholar indexed journals in May 2023. The keywords from our research topic “androgenic alopecia” AND “therapy” were searched on PubMed, which yielded 14,253 results. Following this, we explored the medical subject headings (MeSH) glossary and created a search strategy "Alopecia/drug therapy"[Majr] OR "Alopecia/therapy"[Majr] and were left with 3,998 articles. The inclusion criteria consisted of free full texts published between the 10-year period from 2013 to 2023 in the English language. Only studies done on humans were taken into consideration, which left us with 44 results in the end. Out of those, after quality appraisal, we were left with 35 studies that were included in this review.

## Review

Genetics and AGA

AGA is a complicated condition that is impacted by a combination of environmental and genetic factors. Genes related to the androgen receptor and 5-AR have been identified as significant players in AGA. Recent genome-wide studies have found strong associations between AGA and the androgen receptor gene, as well as the ectodysplasin A2 receptor on the X chromosome [6]. Existing research has identified several genes, such as PGD2, neurotrophin-3 protein, PGDS, BMP2, BDNF, ephrinA3, neural growth factor-β, GSN, and ASS1, to show varying levels of expression in both, scalp biopsies and cell cultures from the dermal papilla, of individuals suffering with AGA; nonetheless, more research is necessary to authenticate these findings [7]. AGA, despite its connection to the activity of androgenic hormones and genetic predisposition, also involves familial clustering. However, it can manifest in multiple family members without following a single-gene inheritance pattern, making it a complex trait [6].

Pathophysiology

**Figure 1:**
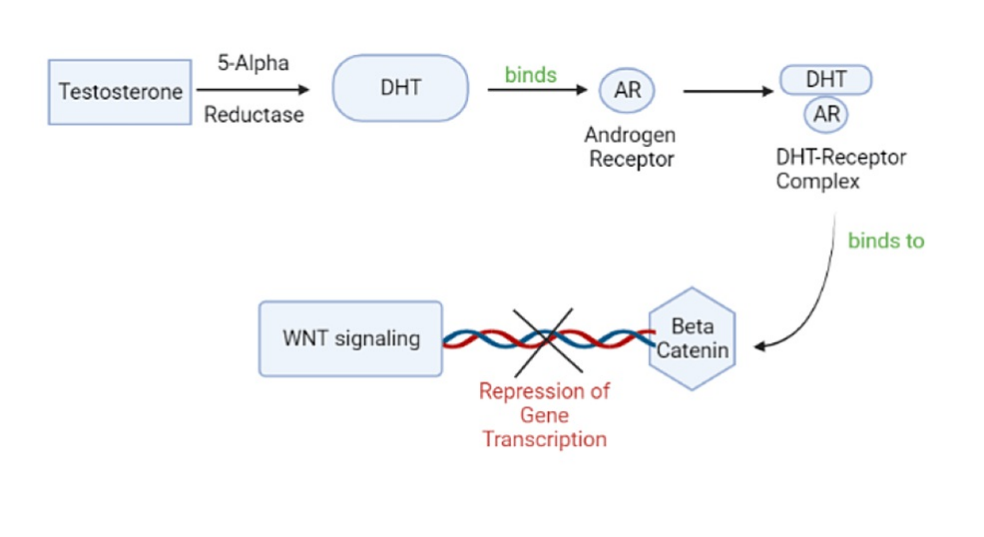
Pathophysiology of AGA AGA: androgenic alopecia, DHT: dihydrotestosterone Created using BioRender.com Image credits: Parth S. Bajoria, Priyansh Patel, and Prathma Anandbhai Dave

The hair follicle experiences three primary stages in its lifecycle. During the anagen (growth) stage, the hair follicle undergoes an active phase where it assumes a structure resembling an onion and functions to generate the hair strand. It is followed by the catagen phase, which lasts for a few weeks, where the hair shaft loses 1/6th of its diameter by apoptosis-driven regression. This is followed by the telogen or dormant phase, where there is no hair growth [8]. AGA occurs due to the gradual reduction in the length of consecutive growth phases of hair (anagen phases) and the gradual shrinking of hair follicles that are genetically susceptible, triggered by androgens. Additionally, the process involves the substitution of thick, dark hairs (terminal hairs) with thin, lightly colored hairs (vellus hair) [9]. The conversion of testosterone to DHT is primarily carried out by the enzyme 5-AR, and DHT plays a crucial role in the development of AGA in males. 5-AR exists in three different forms: types I, II, and III. The skin, encompassing the sebaceous glands and hair follicles, primarily has the type 1 enzyme [10]. In about two-thirds of biopsy cases, there is an observed presence of a relatively moderate perifollicular lymphohistiocytic inflammatory infiltrate surrounding the infundibulum. However, this finding lacks specificity since it is also observed in approximately one-third of normal control cases [11]. A recent development in our understanding of hair follicle regeneration and hair shaft growth involves the recognition of the vital role played by the Wnt/β-catenin signaling pathway in maintaining the properties of dermal papilla cells (DPC) that are necessary for these processes. Additionally, it has been found that androgens and ligand-activated androgen receptors can have a negative impact on the Wnt/β-catenin signaling pathway, as shown in Figure 1. More precisely, increased expression of glycogen synthase kinase 3 beta by androgens is what obstructs this pathway [12]. One distinguishing feature of AGA is the perceived enlargement of sebaceous glands in relation to the size of miniaturized hair follicles. However, it is important to note that this observation is not indicative of true sebaceous hyperplasia, as the sebaceous glands maintain their normal size and structure and their enlargement is only apparent [13]. Another hypothesis regarding AGA suggests that the disappearance of the arrector pili muscle is a contributing factor. The smooth muscle plays a role in regulating body temperature and sebum production while also serving as a link between the hair follicle and the upper layers of the skin. Researchers have put forward the idea that the loss of the arrector pili muscle could be linked to the permanent development of AGA [14].

Consequences of AGA

**Figure 2:**
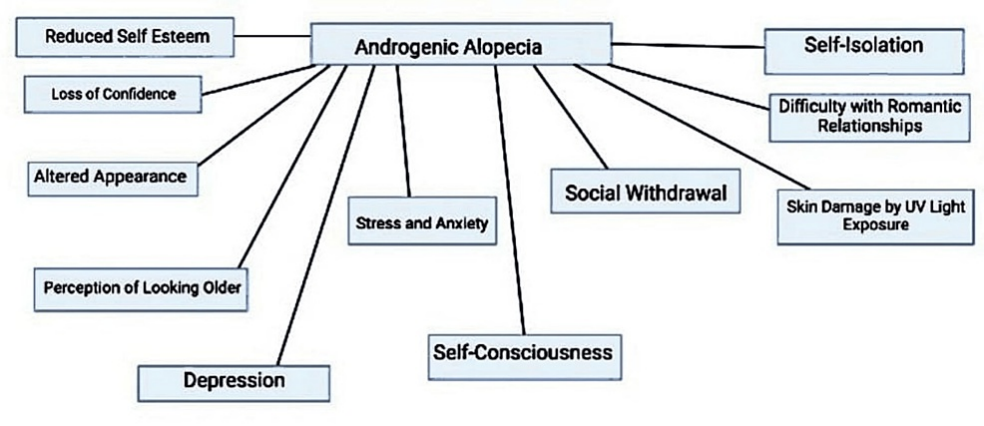
AGA and its consequences AGA: androgenic alopecia, UV: ultraviolet Image credits: Parth S. Bajoria, Priyansh Patel, and Prathma Anandbhai Dave

It is clear that the development of AGA is dependent on factors such as age, lifestyle, and hormonal disorders. AGA tends to affect more individuals with advanced age and stressful lifestyles. Hair is a crucial part of an individual’s appearance, and losing hair can create a feeling of looking older and losing confidence. There are plenty of other psychological effects that follow, which are shown in Figure 2 [15].

Diagnosis

The diagnosis of AGA involves assessing the patient's medical history, clinical findings, duration and location of hair loss, shedding or thinning of hair, and reviewing family history, current medications, and conducting basic laboratory tests to exclude other possible causes of hair loss [3]. Evaluation of AGA is usually done by pull test or trichoscopy. To identify ongoing hair loss, the hair-pull test is conducted by gently pulling 50-60 hairs near the scalp using the middle finger, index finger, and thumb. If six or more hairs are detached during this process, it suggests that there is ongoing hair loss [5]. Trichoscopy is a non-invasive technique used to examine the hair and scalp, which provides valuable information about various characteristics. The most commonly observed trichoscopic features in AGA include variations in hair diameter (anisotrichosis), peripilar signs (brown or white halo surrounding a hair follicle), the presence of yellow or white dots (a marker of severe AGA), a honeycomb pattern of pigmentation (seen in individuals with darker skin tones), bald patches, and arborizing red lines [16].

Classification

**Figure 3:**
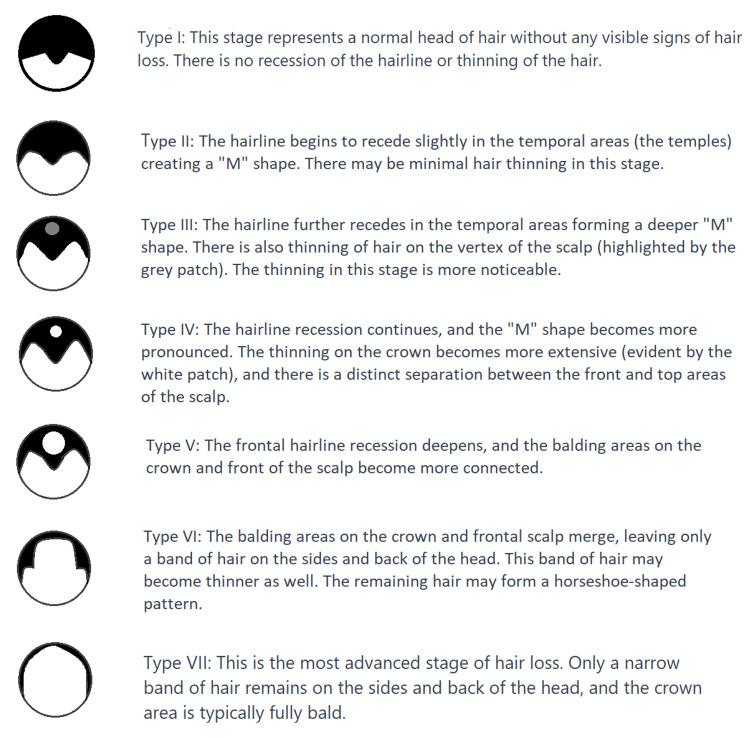
Hamilton-Norwood classification for male-pattern baldness The image was made based on information obtained from Gupta et al. [17], an open-access article distributed under the terms of the Creative Commons Attribution-NonCommercial-ShareAlike 3.0 License, which allows others to remix, tweak, and build upon the work non-commercially, as long as the author is credited and the new creations are licensed under the identical terms. Image credits: Parth S Bajoria, Priyansh Patel, and Prathma Ananbhai Dave

Devised by Dr. James Hamilton and later revised by Dr. O'Tar Norwood, the Hamilton-Norwood classification is a commonly employed system for classifying MPB. It consists of seven stages (types I-VII) that depict the progression of hair loss in men, from minimal to extensive [17]. Figure 3 shows a brief description of each stage.

Treatment

Treating AGA is challenging due to its chronic nature and the interplay of genetic and environmental factors. While the Food and Drug Administration (FDA) has approved only topical minoxidil and oral finasteride, several non-FDA-approved treatments have shown effectiveness. Further research is needed to explore these newer treatments, which are mentioned below [3].

Non-medical Treatment

The most straightforward and convenient approach for addressing mild male AGA is through camouflage. This involves adopting hair styling techniques to conceal the balding scalp, utilizing electrostatically-held small fibers, and dyeing the scalp to match the hair color. These affordable measures can yield cosmetically pleasing outcomes. Additionally, modern wigs offer the ability to style and wash and provide a natural appearance [18].

Medical Treatment

Minoxidil: Topical minoxidil (2% or 5%) was the first FDA-approved drug for the treatment of AGA. It is available as a topical solution as well as foam preparation; however, the foam preparation is associated with lesser local reactions such as erythema or pruritus as per Hasanzadeh et al. [19]. Multiple studies have reported that combination therapy is more efficacious than 5% topical minoxidil alone. Faghihi et al. conducted a study on 50 participants for about 12 months and suggested that adding low-level light therapy to topical minoxidil therapy was more effective than minoxidil therapy alone [20], whereas Pakhomova et al. conducted a study on 69 men divided into three groups of topical minoxidil therapy alone, platelet-rich plasma (PRP) alone and PRP+minoxidil therapy. Her study concluded that combination therapy was highly efficacious as compared to either monotherapy and that PRP monotherapy was more efficacious than topical minoxidil in most aspects [21]. Mirmirani et al. conducted a prospective pilot study that was placebo-controlled and double-blinded. The study evaluated the efficacy of minoxidil topical foam 5% compared to a placebo in 16 men diagnosed with type IV or V AGA and concluded that minoxidil was equally effective in the treatment of both, the frontal and the vertex of the scalp, which are the most commonly affected areas in men with AGA, although there is a variation in regional gene expression profile of these areas [22]. Sakr et al. concluded after a 32-week pilot study that the use of a multimodal minoxidil formulation containing diclofenac and tea tree oil, demonstrated greater efficacy, stability, and safety for treating AGA by promoting earlier responses, potentially due to diclofenac's anti-inflammatory effects on scalp and hair follicles and tea tree oil's ability to eliminate microbial or fungal presence in follicles [23].

5-AR inhibitors: Oral finasteride was the other drug approved by FDA to be used for AGA. To minimize the systemic effects associated with its use, the use of topical finasteride has been suggested in recent years as it selectively targets 5-AR inhibition in the scalp. Piraccini et al. supported this finding with their study on 458 patients although the increase in hair count between oral and topical formulation groups was similar [1]. As with minoxidil, combination therapy generates superior outcomes in patients taking oral finasteride. Van Neste et al. supported this in their study of 22 patients and concluded that the use of oral finasteride along with topical 5% minoxidil (applied at a volume of 1 mL once a day) led to an improvement in the growth of underactive hair follicles although it did not result in an increase in the speed of hair growth [24]. Dutasteride is a second-generation drug of the same class. It shares a similar side-effect profile as finasteride although it is more efficacious as per Shanshanwal et al. [10].

PRP therapy: PRP is derived from whole blood by centrifugation. Gressenberger et al. conducted a pilot study on 30 male patients and said that PRP did not significantly improve hair growth as a monotherapy [25]. Singh et al. studied 80 patients using double-blinded randomized controlled trials (RCTs) and concluded that PRP, along with topical minoxidil 5%, was more efficacious than either PRP or topical minoxidil alone and also PRP demonstrated quicker results compared to topical minoxidil [26] as did Pakhomova et al. suggest [22].

Platelet lysate (PL): PRP and PL are produced in a similar manner, but PL undergoes an additional step that increases the concentration of growth factors in the serum. It is faster acting as compared to PRP. Guan et al. studied 107 AGA patients and said that PL was as efficacious as PRP in terms of increasing hair count, diameter, and absolute anagen counts, making it ideal to replace PRP [27].

Low-level light therapy (LLLT): LLLT or photobiomodulation delivered via caps or helmets has been claimed to prevent MPHL, as well as FPHL. In a 26-week-long study of four RCTs, Jimenez et al. concluded that the increase in terminal hair count by LLLT was similar to the results observed in short-term trials of a 5% minoxidil topical solution and a daily dose of one mg of finasteride, but less effective compared to longer-term trials lasting at least one year [28]. Blum et al. in their study on 119 subjects with the cold X5 hair laser said that chronic therapy leads to hair growth [29].

Miscellaneous Treatments

Treatment for AGA usually continues for an individual’s entire life and therefore taking into account the risk/benefit profile of a drug takes prime importance. Specifically, 0.2% topical caffeine-based solutions are typically safe with very minimal adverse effects for long-term treatment of AGA, and they are not inferior to topical 5% minoxidil therapy as per Dhurat et al. [9]. Prostaglandins (PGs) play a crucial role in the hair follicle cycle, with PGE2-promoting hair growth and PGD2 inhibiting it. Hossein Mostafa et al. compared topical cetirizine 1% (a PGD2 inhibitor) with minoxidil 5% and found that cetirizine had fewer side effects. Both groups showed increased hair density after 16 weeks, but the minoxidil group had greater improvement [30]. Adipose tissue-derived stem cells (ADSCs) release growth hormones that promote cell development, and Tak et al. suggested that applying ADSC-cetirizine solution directly to the scalp, along with finasteride or minoxidil, could be a viable alternative treatment for AGA [31]. Botulinum toxin (BT) derived from Clostridium botulinum blocks acetylcholine release, relaxing muscles, improving blood flow, increasing oxygen, reducing DHT activation, and promoting hair regrowth. Zhou et al. studied the safety and effectiveness of BT type A and concluded that BT type A given along with finasteride has higher efficacy as compared to BT type A injections alone [2]. Pekmezci et al. studied herbal shampoo and solutions containing anti-androgenic, anti-inflammatory, anti-oxidative, and angiogenic properties, on 120 subjects and concluded that they reduced hair loss in patients with AGA. Moreover, the shampoo and solution used together had higher efficacy than either used alone [32]. Procyanidin B2, abundantly present in apples, is a safe and highly effective compound for promoting hair growth and improving skin quality, as supported by Tenore et al.'s clinical trial using AppleMets and laboratory studies showcasing increased keratin content. This natural extract-based nutraceutical supplement enhances hair density, weight, and keratin content simultaneously [33].

Surgical Treatment

Hair transplantation, commonly used for medical treatment failure/resistant AGA, is a surgical procedure that implants new hairs into areas with significant hair loss, providing a natural look and permanent results with over 90% graft survival. It can be classified as follicular unit transplantation (FUT) or follicular unit extraction (FUE) and robotic techniques are also available, albeit with higher costs and maintenance demands [34]. The major treatments are discussed and compared in Table 1.

**Table 1:** Comparing therapeutic modalities for AGA AGA: androgenic alopecia, ATP: adenosine triphosphate, FDA: Food and drug association, mL: milliliter, mg: milligram, nm: nanometer, mW: milliwatt, FUT: follicular unit transplantation, FUE: follicular unit extraction, LLLT: Low-level light therapy, PRP: platelet-rich plasma, 5-AR: 5-alpha reductase

Drug	Mechanism of Action	Dosage and Route	Adverse Effects	Comments
Minoxidil	The Influx of calcium ions up-regulates ATP-synthase, which enhances the process of stem cell differentiation and promotes hair growth [18].	1 mL applied twice a day. Available in 2% and 5% topical as well as foam preparation [3].	Contact/Irritant dermatitis, pruritus, skin irritation and erythema, facial hypertrichosis [34].	First-line FDA-approved therapy. Easy to use. Loss of results on discontinuation [18]. Cost-efficient.
Oral Finasteride	Highly selective and binds irreversibly to type-II 5-AR [3].	1 mg once daily [3].	Erectile dysfunction, reduced libido, orthostatic hypotension, and dizziness [34].	FDA-approved. Good compliance due to a once-daily oral regimen. Cessation of therapy leads to the loss of observed results [19] and the reversal of any side effects that may have occurred [3].
Topical Finasteride	Highly selective and binds irreversibly to type-II 5-AR [3].	0.25% spray or 1% gel [3].	Contact dermatitis, skin irritation, sexual side effects (rarely) [3,18].	Lesser systemic side effects than oral counterparts [18]. Variable response to therapy.
Dutasteride	Acts as an inhibitor of both the iso-enzymes of 5-AR (types I and II) [3].	2.5 or 5 mg once daily [34].	Erectile dysfunction, reduced libido, gynecomastia [18].	Higher efficacy than finasteride [18]. Used off-label.
PRP therapy	Enhances blood flow to the hair follicles, preventing cell death, thereby extending the growth phase [18].	Injections of 4-8 mL via intra-dermal or subcutaneous route. 3-4 sessions monthly, followed by a session every three months [34].	Minor bleeding, pain, and headache [3].	Works better when used as a combination therapy with topical agents [26].
LLLT	Expedites the process of mitosis, stimulates stem cells within the hair follicles, and reduces inflammation [28].	Wavelengths ranging from 650 to 900 nm and a power of 5 mW can be an effective treatment choice [28].	Paresthesia, mild urticaria, and scalp tenderness [3].	Capillus laser cap and Hairmax laser comb are the only 2 FDA-approved devices for AGA as of now [34]. Excellent safety profile.
Hair transplantation	Extraction of hair follicles from the back of the scalp, where hair is immune to balding, and relocating them to the areas affected by the loss of hair [18].	Done with either the FUT or FUE method [18].	Pain, bleeding, pruritus, scarring [3].	The graft survival rate is more than 90% in AGA patients [34]. Generates a natural appearing look. One of the costliest treatments for AGA.

Limitation

Most of the clinical trials were of short duration, and a few were conducted on small sample sizes. Hence, larger and longer studies are further required. We omitted articles in languages other than English and also omitted animal studies. Only articles published in the last decade were considered so there might be other relevant studies that are published before this decade. Only literature found on PubMed was reviewed, and other databases were not explored. Only clinical trials were included to make results in the study. Results from other types of studies were excluded. Moreover, the lack of research evidence regarding the use of nutritional supplements and dietary options in the treatment of AGA and the management of environmental risk factors associated with AGA also needs to be addressed.

## Conclusions

This narrative review examined the current therapeutics available for AGA, putting light on the advancements and challenges in treating this common hair loss condition. Throughout the analysis, it became evident that various treatment options exist, including pharmacological and non-pharmacological approaches. Pharmacological interventions, such as minoxidil and finasteride, have demonstrated efficacy in promoting hair growth and slowing down hair loss in many individuals. However, their effectiveness can vary among different patients, and they may be associated with certain side effects. Moreover, the long-term outcomes and maintenance of hair growth with these medications need further investigation. Non-pharmacological approaches, including LLLT and PRP therapy, have shown promise in stimulating hair growth and improving the overall condition of the scalp. Nevertheless, more rigorous clinical trials are required to determine the potency of these treatments and refine the most-effective treatment protocols. Our article compares the various treatments and also portrays the side-effect profile. We also talked about various combination therapies available and their efficacy demonstrated in different studies. We have also talked about some newer treatments, which have been proven safe but need more extensive research. There is very little research available for the surgical treatment of AGA; hence, extensive research is required for it. Although there are many treatments available, a definitive treatment regimen for AGA remains elusive. Gene therapies, stem cell-based treatments, and novel pharmaceutical agents currently under investigation hold great potential for revolutionizing the management of this condition. Nevertheless, these emerging therapies necessitate rigorous evaluation to ensure safety, efficacy, and long-term outcomes.
